# On the directionality of membrane coupled Helmholtz resonators under open air conditions

**DOI:** 10.1038/s41598-024-79568-9

**Published:** 2024-11-13

**Authors:** R. Domingo-Roca, A. Feeney, J. F. C. Windmill, J. C. Jackson-Camargo

**Affiliations:** 1 Centre for Ultrasonic Engineering, Electronic & Electrical Engineering, University of Strathclyde, Glasgow, UK; 2 Centre for Medical and Industrial Ultrasonics, James Watt School of Engineering, University of Glasgow, Glasgow, UK

**Keywords:** Acoustic metamaterials, Directionality, Helmholtz resonators, Membranes, 3D-printing, Low-frequency sound absorption, Acoustics, Engineering

## Abstract

Controlling the absorption and diffusion of sound in the audible range constitutes an exciting field of research. Acoustic absorbers and diffusers perform extraordinarily well at high frequencies with sizes comparable to the wavelength of the working frequency. Conversely, efficient low-frequency attenuators demand large volumes leading to unpractical sizes, and there is now interest in determining whether the size of the resonator can be reduced while not compromising – or perhaps even decreasing – the working frequency. One viable approach is through the use of metamaterials to enable the control of device dynamics such that heavy sub-wavelength attenuation can be efficiently realised. To achieve this goal, the theoretical (including a mathematical model and the use of finite element analysis) and experimental characterisation of 3D-printed membrane-coupled Helmholtz resonator (HR) acoustic metamaterials (AMMs) is explored. The results reveal good agreement between theory and experiments, and show that membrane-coupled HR AMMs feature heavy sub-wavelength acoustic attenuation (*λ*/55) while also showcasing directional responses under open air conditions. These features are explained by the interplay between resonator size, membrane characteristics, and the presence of two acoustic ports. It is anticipated that, together with recent advances on smart AMMs, these systems will foster new progress in the development of dynamic AMMs for wideband attenuation.

**Supplementary Information:**

The online version contains supplementary material available at 10.1038/s41598-024-79568-9.

## Introduction

It has been long appreciated that exposure to persistent noise delivered above 115 dB sound pressure levels (SPL) can damage the inner ear and induce significant pathological damage in the central auditory nervous system^1–3^. Noise-induced hearing loss can occur due to one-time exposure to an intense pulse of sound, or by long-term exposure to sound levels above 65 dB^4^ – the definition of noise by the World Health Organisation. Hence, low-frequency sound attenuation is of long-standing interest to science and society, whether to achieve non-reciprocal wave propagation, to insulate interior spaces from exterior noise, or for noise cancellation in multiple technological applications such as personal electronics. Conventional approaches aiming to mitigate noise transmission rely on the mass density law principle^5,6^, which has limitations in terms of mechanical properties and thickness of the materials that can be used, and lead to unpractical sizes and cost-inefficient devices. Drawbacks relating to low-frequency attenuation have been addressed over the years via mass-spring-mass models combining multiple materials and degrees of freedom^7,8^, the use of porous materials and microperforated panels^9,10^, and by exploiting thermoviscous acoustic energy dissipation in foams^11,12^. More recent approaches for noise control include the use of metamaterials since they enable sub-wavelength attenuation as a result of their negative bulk modulus, density, and chirality^13,14^, enabling attenuation of low-frequency sound via sub-wavelength constituents whose collective behaviour allows unusual macroscopic wave propagation. At the diffraction regime, the periodic configuration of metamaterials presents bandgaps at wavelengths of the order of the periodicity of the lattice – a feature that has been thoroughly exploited for noise control^15,16^, sound absorption^16,17^, selective filtering^18,19^, and frequency tuning^20,21^. Multitude of configurations have been investigated as acoustic metamaterials (AMMs) for this purpose, including fractal lattices^6,22,23^, decorated membrane resonators^24–26^, Fabry-Perot resonators^27–29^, Helmholtz resonators^30–33^, and their miscellaneous combinations and variations^13,34^. Soft compliant materials and structural members are widely utilised as constituents in metamaterials to introduce coupling mechanisms to match the impedance between the acoustic fluid and the metamaterial and provide near-perfect absorption of sound^5,35^. Nevertheless, these concepts are effective for attenuating single-frequency waves such that broadband attenuation requires complex assemblies or multiple resonators^35^. Over the past two decades, huge progress has been made in the fundamental research of AMMs, which has promoted the development of modern acoustics while also revealing their great potential for engineering applications. Yet, there remain a series of challenges associated with their topological and morphological complexity, achieving wideband attenuation, and their miniaturisation (without compromising their operating frequency) that must be addressed for their optimal application in real-world scenarios.

While a huge variety of resonators have been proposed as AMMs in the literature to tackle these drawbacks^6–9,17,21,22,27^, Helmholtz resonator (HR)-based AMMs, in particular, have attracted the attention of researchers due to their potential for lightweight, broadband low-frequency (below 1,000 Hz) sound manipulation^36–38^. In fact, modifications to traditional HRs have achieved negative dynamic modulus, low-frequency wave absorption, and multifrequency attenuation by using embedded soft structural members^39–44^. Among the many strategies by which sound absorption in metamaterials can be tailored and enhanced, integration of soft structures enables novel interactions between material behaviour and acoustic-wave propagation^5,35,45^. One such approach is to replace the bottom rigid wall of the HR by a membrane. These systems have shown – both theoretically and experimentally – to improve sound transmission loss in the low-frequency regime while also achieving broadband attenuation^13,24,46,47^. Yet, there is a lack of knowledge regarding the parameters that may be influencing the response of the system under open air conditions, leading to an incomplete view of their fundamental behaviour in real-World scenarios, and hindering their full range of applicability. Conventional acoustic testing of membrane-coupled HR AMMs in impedance tubes provides an initial idea of their acoustic behaviour, but limits its complete physical understanding since it considers them as one-port – rather than two-port – acoustic systems. In this paper, membrane-coupled HR AMMs are designed, fabricated, and tested under open air conditions to obtain a more detailed fundamental understanding of their acoustic response. By using this approach, we achieve sub-wavelength, directional acoustic responses of the membrane-coupled system. The paper details the theory needed to characterise the acousto-mechanical response of the system, which is supported by finite element analysis (FEA) and experimental validation.

## Results

### Acoustic response of the Helmholtz resonator AMMs

**Fig. 1:**
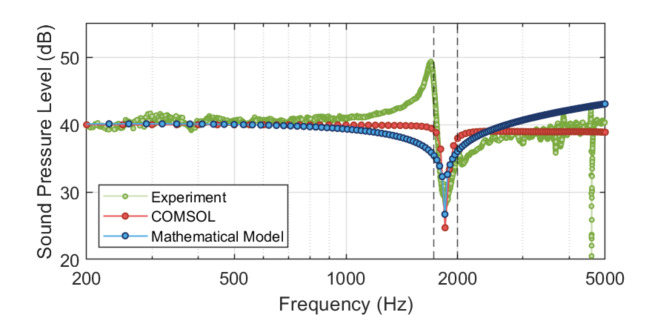
Sound pressure level (SPL, in dB) of a Helmholtz resonator (HR) acoustic metamaterial (AMM) according to the proposed mathematical model (Eq. 4, blue), finite element analysis (FEA, red), and experimental results (green). The two vertical dashed black lines indicate the lower and higher expected bandgap frequencies of the HR according to Eq. (1) – depending on the end-correction factor used. HR AMM dimensions: cavity height (*H*_*c*_) 10 mm, cavity radius (*R*_*c*_) 5 mm, neck height (*H*_*n*_) 2 mm, and neck radius (*R*_*n*_) 1 mm.

Initial validation of the proposed methodology was performed by modelling the HR AMM as a mass-spring system (see Eq. (4), Methods) in the low-frequency regime, where the dimensions of the neck and cavity are the only parameters that dictate the resonant response. Figure 1 shows the acoustic response (sound pressure level, SPL, in dB) of a HR AMM with cavity height (*H*_*c*_) of 10 mm, cavity radius (*R*_*c*_) of 5 mm, neck height (*H*_*n*_) of 2 mm, and neck radius (*R*_*n*_) of 1 mm according to the mathematical model (Eq. (4), blue), finite element analysis (FEA, red), and experimental results (green). The two dashed vertical black lines indicate the Helmholtz resonances obtained using Eq. (1) at end correction factors of 1.2 and 1.7. This leads to a conventional Helmholtz resonance located between 1726.29 Hz and 1993.35 Hz. The proposed mathematical model predicts the bandgap frequency at 1847.6 Hz, the FEA bandgap is obtained at 1850 Hz, and the experimental results display the bandgap at 1856.25 Hz, showing great agreement between the three approaches. The sound pressure level (SPL) and full width at half maximum (FWHM) are shown in Table 1.

**Table 1:** Acoustic response (includes bandgap frequency, sound pressure level (SPL), and full width at half maximum (FWHM)) of the Helmholtz resonator (HR) acoustic metamaterial (AMM) obtained from the mathematical model, finite element analysis (FEA), and experimental results.

	Bandgap frequency (Hz)	SPL (dB)	FWHM (Hz)
Helmholtz resonator acoustic metamaterial			
Mathematical model	1847.6	28.78	59.92
Finite element analysis	1850	24.70	100
Experimental results	1856.25	28.19	117.19

### Acoustic response of the membrane-coupled HR AMMs

**Fig. 2:**
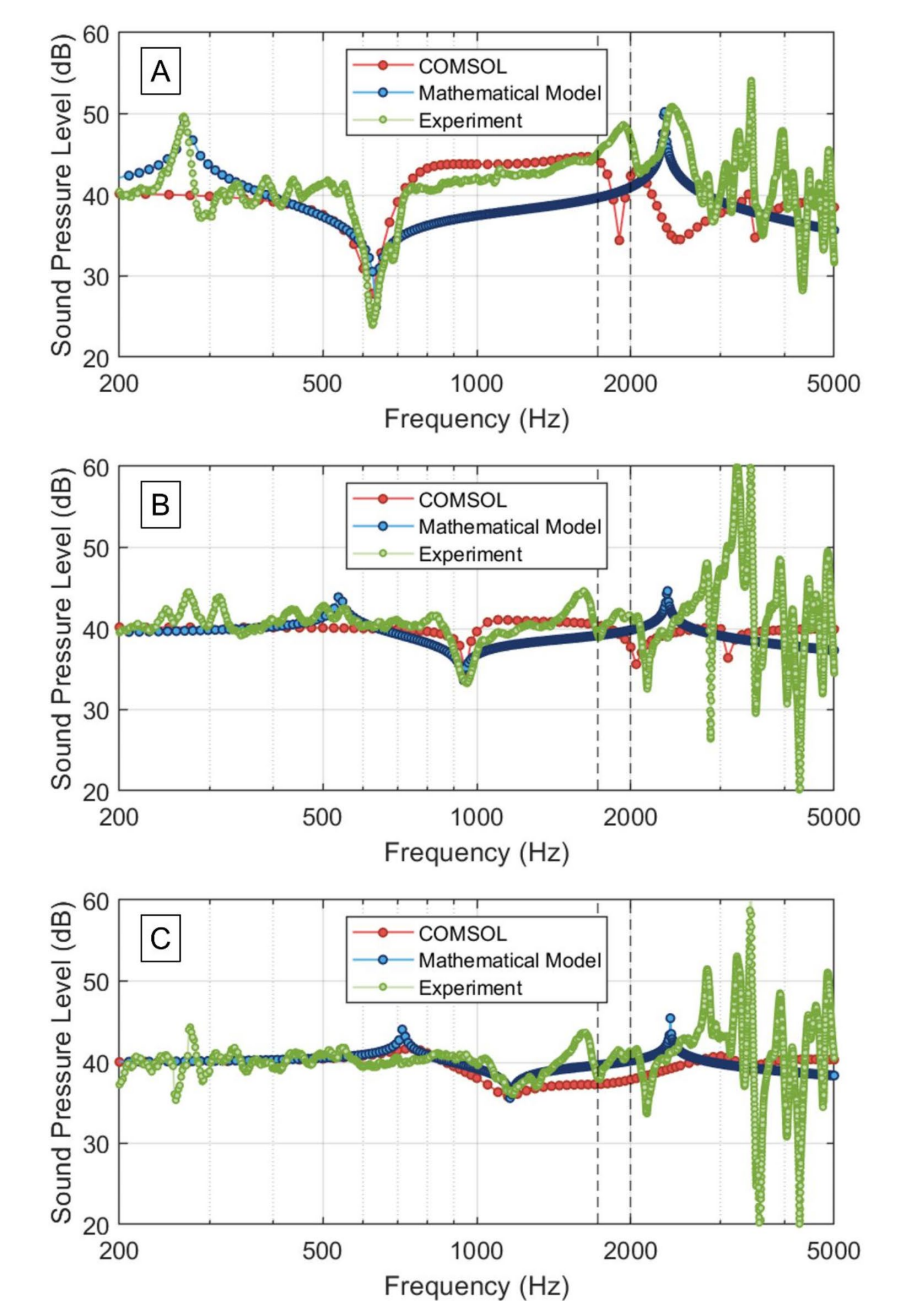
Sound pressure level (SPL, in dB) of the membrane-coupled Helmholtz resonator (HR) acoustic metamaterial (AMM) according to the proposed mathematical model (blue), finite element analysis (FEA, red), and experimental results (green) at membrane tensions of (**A**) 3.37 N/m, (**B**) 10.08 N/m, and (**C**) 18.77 N/m. The vertical dashed lines indicate the lower and higher expected bandgap frequencies of a conventional HR of cavity height (*H*_*c*_) 10 mm, cavity radius (*R*_*c*_) 5 mm, neck height (*H*_*n*_) 2 mm, and neck radius (*R*_*n*_) 1 mm.

After initial validation of the proposed methodology, the membrane-coupled HR AMM system was investigated using Eq. (7) and Eq. (8). These equations introduce equivalent lumped parameters coupling the interaction of the membrane with both the cavity and neck of the HR while also considering the unit cell of the AMM as a two-port acoustic system. Figure 2 (mathematical model in blue, FEA in red, and experiments in green) shows the acoustic response (sound pressure level, SPL, in dB) of the membrane-coupled HR AMMs at applied tensions of 3.37 N/m (Fig. 2A), 10.08 N/m (Fig. 2B), and 18.77 N/m (Fig. 2C). These values were chosen as representative of the full range that was experimentally tested (Supplementary Table S1). The two dashed black vertical lines display the minimum and maximum predicted bandgap frequency of a HR of the same dimensions, as reported in Fig. 1. Figure 2 shows that the bandgap frequency of the membrane-coupled HR AMM can be accurately predicted using the proposed mathematical model, as confirmed by finite element analysis, and as reported in the values from Table 2.

**Table 2:** Acoustic response (includes bandgap frequency, sound pressure level (SPL), and full width at half maximum (FWHM)) of the membrane-coupled Helmholtz resonator (HR) acoustic metamaterial (AMM) at three membrane tension values (3.37 N/m, 10.08 N/m, and 18.77 N/m) obtained from the mathematical model, finite element analysis (FEA), and experimental results.

	Bandgap frequency (Hz)	SPL (dB)	FWHM (Hz)
*T* = 3.37 N/m			
Mathematical model	635.27	23.35	39.74
Finite element analysis	630	23.53	32.98
Experimental results	626.56	23.86	68.87
*T* = 10.08 N/m			
Mathematical model	942.79	33.58	50.38
Finite element analysis	950	34.55	43.25
Experimental results	956.25	33.26	91.82
*T* = 18.77 N/m			
Mathematical model	1151.10	35.44	91.11
Finite element analysis	1150	35.76	1032.18
Experimental results	1178.12	36.03	212.56

### Influence of tension on the acoustic response of the membrane-coupled HR AMMs

**Fig. 3:**
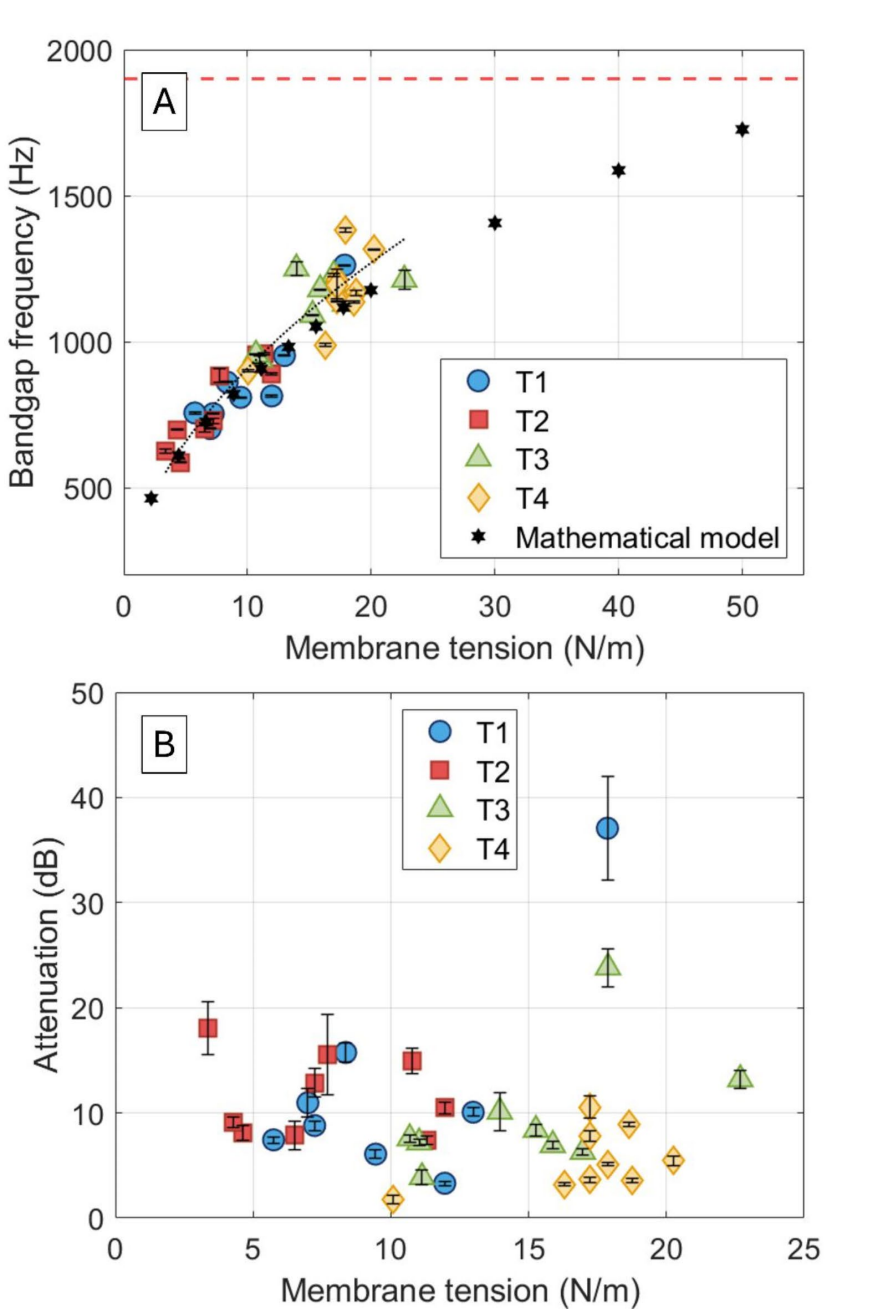
(**A**) Bandgap frequency of the membrane-coupled Helmholtz resonator (HR) acoustic metamaterial (AMM) as a function of the applied tension on the membrane. T1-T4 show four sets of applied tensions, the black hexagrams show the results obtained applying the proposed mathematical model, and the dotted black line shows the fitting of the experimental data (with R^2^ = 0.9040). The dashed red line displays the bandgap frequency of a HR AMM of cavity height (*H*_*c*_) 10 mm, cavity radius (*R*_*c*_) 5 mm, neck height (*H*_*n*_) 2 mm, and neck radius (*R*_*n*_) 1 mm. (**B**) Acoustic attenuation (in dB) of the membrane-coupled HR AMMs as a function of the applied tension on the membrane. Error bars represent standard deviation with *N* = 6.

It is clear from Fig. 2 that the tension applied on the membrane has a non-negligible influence on the acoustic response of the membrane-coupled HR AMMs. To better understand this dependency, the bandgap frequency (Fig. 3A) and the acoustic attenuation (Fig. 3B) of the membrane-coupled HR AMMs were measured and plotted against the applied membrane tension.

### Directionality of membrane-coupled HR AMMs

**Fig. 4:**
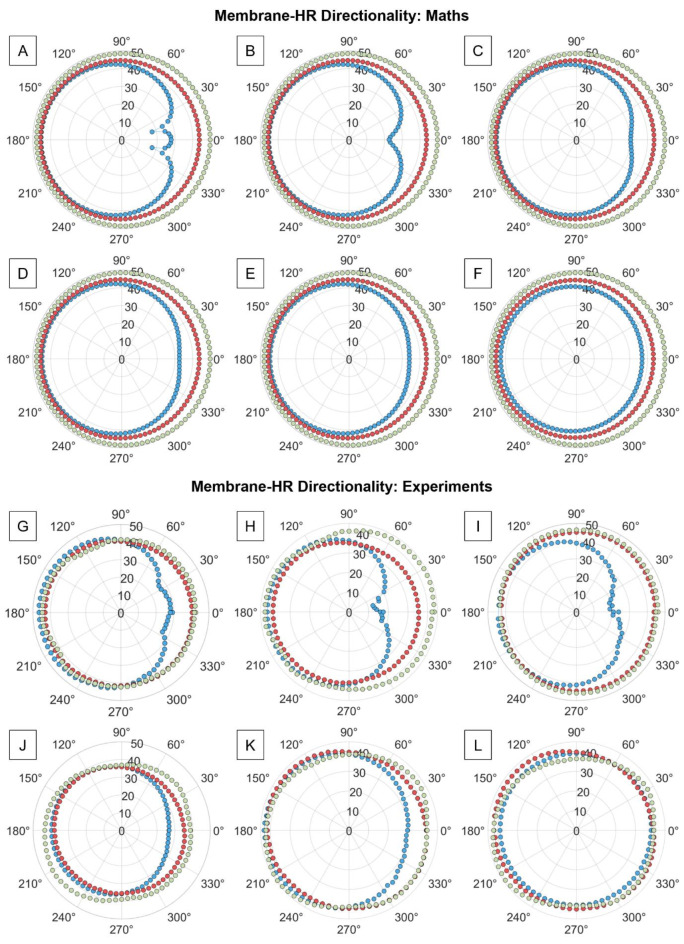
Directional response of a membrane-coupled Helmholtz resonator (HR) acoustic metamaterial (AMM) (**A**)-(**F**) according to the proposed mathematical model, and (**G**)-(**L**) from experimental data at increasing membrane tension. The red and green data points correspond, respectively, to 1850 Hz and 3000 Hz. The blue data points correspond to the bandgap frequency of each system, dictated by the applied membrane tension: (**A**) 700.6 Hz, (**B**) 745.4 Hz, (**C**) 787.0 Hz, (**D**) 827.0 Hz, (**E**) 1001 Hz, (**F**) 1274 Hz. (**G**) 702.6 Hz, (**H**) 729.0 Hz, (**I**) 625 Hz, (**J**) 952 Hz, (**K**) 959 Hz, and (**L**) 1136 Hz. Cavity height (*H*_*c*_) 10 mm, cavity radius (*R*_*c*_) 5 mm, neck height (*H*_*n*_) 2 mm, neck radius (*R*_*n*_) 1 mm, and membrane thickness (*h*_*m*_) 60 μm.

The directional response of the membrane-coupled HR AMMs was theoretically (Fig. 4A–F) and experimentally (Fig. 4G–L) investigated as a function of the applied tension on the membrane. The blue, red, and green data points correspond to the centre bandgap frequency of the membrane-coupled HR AMM system, the HR resonance (1850 Hz), and 3000 Hz (arbitrarily chosen to show the behaviour of the system at higher frequencies). Further detail on spectral modifications with respect to the angle of incidence of the acoustic wave are shown in the Supplementary Information (Supplementary Fig. S2, and Supplementary Fig. S5). Figure 4 shows good agreement between the experimental data and the proposed mathematical model, indicating that – for a given resonator size – at low tension values, the acoustic response of the membrane-coupled HR AMMs is cardioid. As membrane tension is increased, the response shifts to display an omnidirectional pattern, a behaviour that is also predicted by the proposed mathematical model.

## Discussion

This work reports the design, mathematical modelling, fabrication, and testing of a series of membrane-coupled HR AMMs under multiple membrane tensions for sub-wavelength acoustic attenuation in open air conditions. The results reported in this study demonstrate the effectiveness of the proposed mathematical model in predicting the acoustic behaviour of HR AMMs both with and without membrane coupling. The accuracy of the model is confirmed through strong agreement with finite element analysis and experimental results including various membrane tensions.

**Fig. 5:**
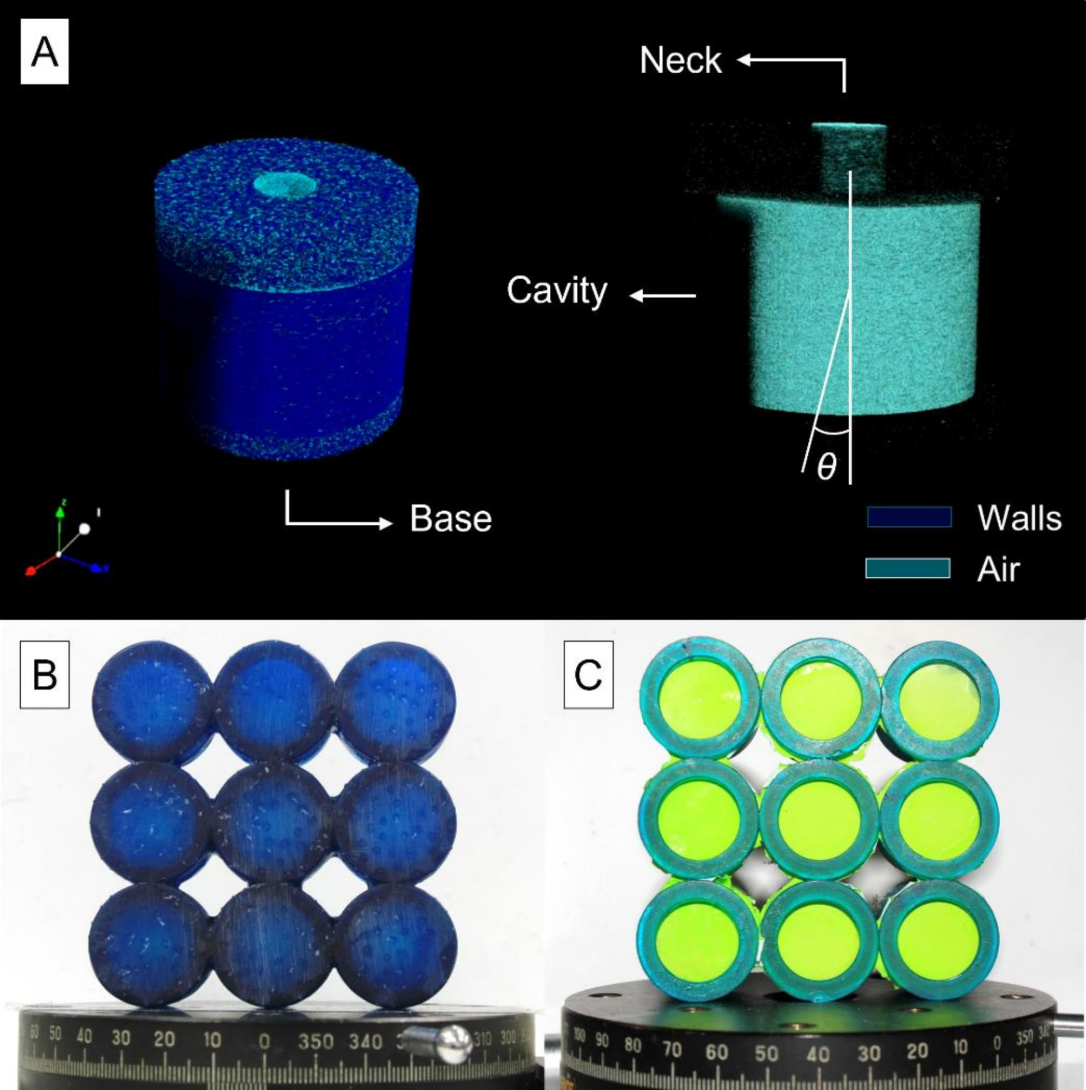
(**A**) Representation of the geometry of a Helmholtz resonator (HR), indicating its multiple components (neck, cavity, and base – explicitly mentioned because this is replaced by a membrane), and indicating in different shades of blue the walls and the air inside the resonator. The definition of the angle of incidence (*θ*) of the acoustic field is shown in the right-hand-side panel of (**A**), leading to *θ* = 0° when the pressure field impinges directly onto the base (or membrane) of the HR. Representations obtained via X-ray micro-computed-tomography (details in Supplementary Material). (**B**) and (**C**) show, respectively, the 3D-printed HR and membrane-coupled HR acoustic metamaterials from the front view (*θ* = 0°). (**C**) Also shows the 3D-printed rings that sit on top of the membrane. Scale bars, 10 mm.

Fabrication of the HR AMM was successfully achieved via stereolithography 3D-printing using commercially available materials. Manufacture of the membrane-coupled HR AMMs was realised by 3D-printing of base-free HR AMMs and the posterior attachment of pre-stretched 60 μm nitrile membranes. The membranes were finally clamped to the base of the HR via ring structures (see Fig. 5). Four sets of tensions were investigated in this manner, although they presented more variability than initially anticipated.

The initial validation of the mathematical model, based on the HR AMM without membrane coupling, showed excellent agreement with both FEA and experimental data. The predicted bandgap frequency (1847.6 Hz) was closely matched by the FEA (1850 Hz) and experimental results (1856.25 Hz), indicating that the model effectively captures the primary acoustic response of the system under open air conditions, including a good prediction of the sound pressure level (SPL). However, some discrepancies were noted in full width at half maximum (FWHM) between the mathematical model (59.92 Hz), and FEA and experiments (100 Hz and 117.19, respectively). While the discrepancies between experiments and FEA are mainly attributed to the FEA solver resolution (25 Hz), the bigger differences with the mathematical model suggest that additional factors such as structural damping or nonlinear effects that broaden the resonance peak might be lacking. One of these factors could be the introduction of wall deflection, which has proven to influence the acoustic response of the AMM when using elastic materials^35^. Although this work reports the fabrication of AMMs using rigid plastics, the low thickness of the walls (1 mm) could be influencing the bandwidth.

Introducing a tensioned membrane to the HR AMM system adds a degree of complexity. Membrane AMMs have been examined in the past and have revealed a similar behaviour to that reported here^25,26^. In membrane AMMs, sound attenuation is uniquely dictated by the eigenmodes of the membrane, whereas in membrane-coupled HR AMMs, sound attenuation arises as a result of the interaction of the external plane wave excitation, the inner acoustic pressure, and the membrane. The mathematical model accurately predicts the increase in bandgap frequency with increasing membrane tension, as demonstrated across all tension levels tested (3.37 N/m, 10.08 N/m, and 18.77 N/m, as displayed in Fig. 2). The mathematical model and the experiments show excellent agreement in terms of predicted bandgap frequency and SPL. However, the experimental FWHM values were significantly broader than those predicted by the model, particularly at higher membrane tension values. The membrane’s nonlinear mechanical response under high tension could lead to modifications in FWHM as a consequence of increased damping effects as the membrane tension rises, possibly due to frictional losses or viscoelastic effects within the membrane material. These effects are not fully captured either in the mathematical model nor in FEA. Figure 2 suggests to support this hypothesis, since it can be observed that at low tension values (Fig. 2A, B), the first membrane resonance is well predicted by the mathematical model, which results in sound amplification due to its resonance.

Figure 3A further shows that the mathematical model accurately predicts the bandgap frequency of the membrane-coupled HR AMMs. By solving Eq. (7) and Eq. (8) for frequency as a function of membrane tension, *T*_*s*_, it can be found that in membrane-coupled HR AMMs, the bandgap frequency response is proportional to $\:\sqrt{C+{T}_{s}-\sqrt{1-{T}_{s}+{{T}_{s}}^{2}}}$. This expression is used to fit the experimetnal data, leading to a coefficient of determination of *R*^*2*^ = 0.9040 (Fig. 3A). Both theoretical and experimental results suggest that, as membrane tension increases, the maximum bandgap frequency will be determined by the dimensions of the HR, therefore suggesting that a conventional HR can be thought of as a membrane-coupled HR system with infinite applied tension. In ideal theoretical models the first eigenfrequency of the membrane carries the most energy, leading to increased acoustic power that can be transmitted to the cavity. Nevertheless, in experimental settings of membranes with uneven or uncontrolled tension, this energy transmission can be fairly inefficient. These variations in membrane tension cause direct propagation of acoustic radiation through the structure, which decreases acoustic attenuation^48^ – as a consequence of an increase in stiffness – leading to less re-radiated energy at higher tensions. The experimental observation of decreasing sound attenuation with increasing membrane tension, as shown in Fig. 3B, aligns with the model’s predictions. The decrease in attenuation suggests that higher tensions reduce the membrane’s ability to effectively trap and dissipate acoustic energy, potentially due to reduced membrane compliance. The presence of outliers in the experimental data might be related to inconsistencies in membrane tension or inhomogeneities in the membrane material, which could cause localised variations in acoustic response.

The directional response analysis further supports the model’s predictive capability. The shift from a cardioid pattern at low frequencies to an omnidirectional response at higher membrane tensions was accurately captured by the model and confirmed experimentally. This shift indicates that the membrane’s tension significantly influences the directionality of sound propagation. Membrane-coupled HR AMMs feature two acoustic ports when acoustically driven in open air conditions. As such, it is expected that as the external plane wave impinges on the system from different angles, its interaction with the membrane will vary, directly affecting its mechano-acoustic response and leading to different acoustic patterns. This is not observed in conventional HR AMMs (Supplementary Fig. S2 and S3), and it is explained by the presence of one unique acoustic port through which sound enters the cavity. Once the membrane is introduced, a non-negligible pressure enters the cavity through the membrane end (Supplementary Fig. S4, FEA simulation), which is observed to decrease as *T*_*s*_ increases, resulting to lower mechanical energy flux as *T*_*s*_ increases. This characteristic feature of membrane-coupled HRs leads them to display a directional response, which is mainly dictated by the size of the resonator itself and the applied membrane tension (Fig. 4, and Supplementary Fig. S5 and S6). For a membrane-coupled HR AMM under 20 N/m tension, the acoustic response at resonance is nearly-omnidirectional (Fig. 4L). If both the neck and cavity height are halved, a cardioid response is obtained at a same tension value (Supplementary Fig. S7), a feature that could be exploited for miniaturisation purposes. Moreover, membrane tension was also observed to play a role in the directionality of the system; as *T*_*s*_ increases, the membrane-coupled HR AMMs bandgap approaches that of an unmodified HR, and the directional response of the system shifts from cardioid to omnidirectional (Fig. 4) at the bandgap centre frequency. One could think that directionality arises from the unique interaction of sound with a membrane, since such a system also contains two acoustic ports. This was theoretically and experimentally investigated (Supplementary Fig. S8); a uniformly tensed membrane under acoustic excitation only contains the impedance of the membrane. While sound interacts with both sides of the membrane, the acoustic flow on each side is equal in magnitude but in opposite directions. This confirmed that the directional response of the membrane-coupled HR AMMs arises from the interaction of the acoustic field with the two ports of the system^49^.

In summary, this work reports a mathematical model, FEA simulation, fabrication, and experimental testing of membrane-coupled HR AMMs to determine their acoustic response in open air conditions. The main focus of this work is to understand the role of the membrane on the acoustic response of the AMM and obtain better fundamental knowledge of its physical response in a manner that has not been previously investigated. SPL attenuations of 20 dB were measured in HR AMM devices, in line with similar studies investigating larger samples^13,24–26,48,50^. In membrane-coupled HR AMMs, acoustic attenuation was measured to decrease with increasing membrane tension due to increased sound transmission through the structure. The frequency response of these systems is dictated by membrane tension, leading to sub-wavelength attenuations of *λ*/55 and providing good agreement between theory and experiments. While the bandgap frequency shift is dictated by membrane tension, the directional response arises as a combination of membrane tension (particularly important at high tension values, making the system behave, effectively, as a one-port system) and unit cell size. This work provides exciting results towards the development of directional AMMs for audio sound attenuation.

The proposed mathematical model provides a robust framework for predicting the acoustic behaviour of both standard and membrane-coupled HR AMMs. While the model effectively predicts key features such as bandgap frequency, attenuation levels, and directional response, some discrepancies are observed in FWHM, particularly at higher membrane tensions, suggesting that the model can be further refined to account for nonlinear effects and material-specific damping mechanisms. Future work should focus on incorporating these factors into the model, as well as exploring the impact of membrane material properties on the acoustic response to enhance applicability in the design of advanced acoustic metamaterials.

It is anticipated that this work, together with recent advances in smart (meta)materials could be applied to the development of novel smart AMMs. For instance, one could actively increase membrane tension to make the system display an omnidirectional response at a given frequency, or to attenuate noise coming from a particular direction, and find valuable applications in military and defence, consumer electronics, and environmental noise control. In military and defence applications, the directional acoustic response could aid in designing advanced sound stealth technologies, enhancing sound masking and detection systems. The ability to adjust membrane tension to modulate directional sound attenuation can be leveraged in consumer electronics, such as noise-cancelling headphones and soundproofing system, s where tailored acoustic performance is crucial. Additionally, the development of membrane-coupled HR AMMs could contribute to more effective environmental noise control in urban settings, offering sub-wavelength attenuation for compact noise barriers. Moreover, we are confident that the recent advances in functional, active 3D-printable materials (such as electro- and magneto-active materials), will enable multi-material 3D-printing of active membrane-coupled AMMs such that assembly processes are minimised.

## Methods

### Mathematical model

A Helmholtz resonator (HR), first proposed by Hermann von Helmholtz in 1862^51^, consists of a rigid-walled cavity connected to a neck through which sound impinges the system (bottles are daily examples of HRs), and its resonance (*f*_*HR*_) uniquely depends on its geometrical characteristics (Eq. 1).

1 $$\:{f}_{HR}=\frac{{c}_{0}}{2\pi\:}\sqrt{\frac{{S}_{n}}{L{\prime\:}{V}_{c}}}$$

where *c*_*0*_ is the speed of sound in the propagating medium, *S*_*n*_ is the cross-sectional area of the neck, $\:L{\prime\:}$ is the extended length of the neck (defined by $\:L{\prime\:}$ = *H*_*n*_ + 1.7*R*_*n*_, where *H*_*n*_ and *R*_*n*_ are the height and radius of the neck, respectively), and *V*_*c*_ is the volume of the cavity (determined by the radius and height of the cavity, *R*_*c*_*and H*_*c*_, respectively) – for a cylindrical resonator.

Multiple authors have investigated membrane-coupled HR AMMs and have proposed mathematical models describing their behaviour^46,52,53^. These models predict the frequency response of the system, but they disregard the fact that membrane-coupled HR AMMs consist of two acoustic ports that allow acoustic pressure to enter the cavity^54^. Under such circumstances, the cavity acts as a compliant reservoir rather than as a pipe connecting the two acoustic ports, which is crucial when the dimensions of the cavity are no longer small compared to the sound wavelength they interact with^54^.

The governing equation of motion for a circular membrane of radius *R*_*m*_, areal density *ρ*_*a*_, and tension *T*_*s*_, in polar coordinates, is given by Eq. (2)^46^.

2 $$\:\frac{{\partial\:}^{2}w\left(r,t\right)}{\partial\:{r}^{2}}+\frac{1}{r}\frac{\partial\:w\left(r,t\right)}{\partial\:r}-\frac{1}{{c}_{m}}\frac{{\partial\:}^{2}w\left(r,t\right)}{\partial\:{t}^{2}}-\frac{{c}_{d}}{{T}_{s}}\frac{\partial\:w\left(r,t\right)}{\partial\:t}=\frac{-{p}_{0}}{{\rho\:}_{a}{c}^{2}}$$

where *w(r*,* t)* is the deflection of the membrane (as a function of the radial position, *r*, and time, *t*), *c*_*m*_ is the speed of sound of the membrane (given by $\:{c}_{m}=\sqrt{{T}_{s}/{\rho\:}_{a}}$), *p*_*0*_ is the uniform pressure distributed over the surface of the membrane, and *c*_*d*_ is the viscous damping. Assuming periodic harmonic excitation, and considering the kinetic energy of an infinitesimal annular element of the displacement in the *n*^th^ mode, it is possible to obtain the displacement of the membrane (Eq. 3)^46^.

3 $$\:w\left(r,t\right)={A}_{n}{J}_{0}\left(\frac{{\mu\:}_{n}}{{R}_{m}}r\right)\text{c}\text{o}\text{s}\left({\omega\:}_{n}t\right)$$

where *A*_*n*_ is an arbitrary constant, *J*_*0*_*(x)* is the Bessel function of the first kind of order 0 ($\:{k}_{n}={{\omega\:}_{n}/c}_{m}$, where $\:{\omega\:}_{n}$ is the eigenfrequency of the *n*^th^ mode of the membrane), and $\:{\mu\:}_{n}$ is the *n*^th^ root to $\:{J}_{0}\left(\mu\:\right)=0$. It must be noted that only axisymmetric vibration modes are considered under uniform pressure excitation, and as such, the ‘*n*^th^ mode’ refers to the ‘*n*^th^ axisymmetric mode’ from here onwards. By averaging the kinetic energy of the membrane over a period *T*, its equivalent mass can be found to be $\:{M}_{eq,m}^{\left(p\right)}={\mu\:}_{n}^{2}\pi\:{R}_{m}^{2}{\rho\:}_{a}/4$^46^. The equivalent stiffness and damping coefficient of the membrane are, respectively, $\:{K}_{eq,m}^{\left(p\right)}={M}_{eq,m}^{\left(p\right)}{\omega\:}_{n}^{2}$ and $\:{D}_{eq,m}^{\left(p\right)}=2\:{\xi\:}_{n}{M}_{eq,m}^{\left(p\right)}{\omega\:}_{n}$ (with $\:{\xi\:}_{n}={c}_{d}/2{\rho\:}_{a}{\omega\:}_{n}$)^46^. This model considers the membrane to behave as a piston where, as detailed by Hu et al.^46^, to ensure that the volume displacements of the membrane and equivalent piston are the same, the space-averaged deflection is taken to equate the displacement of the piston, where the effective force-bearing area of the piston is equal to the area of the membrane. If one considers that the equivalent lumped parameters are instead concentrated at the centre of the membrane, these become $\:{M}_{eq,m}^{\left(c\right)}=\pi\:{R}_{m}^{2}{\rho\:}_{a}{J}_{1}^{2}\left({\mu\:}_{n}\right)$, $\:{K}_{eq,m}^{\left(c\right)}={M}_{eq,m}^{\left(c\right)}{\omega\:}_{n}^{2}$, and $\:{D}_{eq,m}^{\left(c\right)}=2{\xi\:}_{n}{M}_{eq,m}^{\left(c\right)}{\omega\:}_{n}^{2}$^46^, and the model is referred to as the centre-mass model. In this case, the force-bearing area is mode dependent and does not equate to the area of the membrane^46^ (see Supplementary Material).

It is well established that HR systems can be modelled as a mass-spring system in the low frequency regime, which behaviour is described by Eq. (4).

4 $$\:{M}_{1}{\dot{U}}_{1}+{R}_{1}{U}_{1}+{K}_{1}\int\:{U}_{1}dt={p}_{0}$$

where $\:{M}_{1}={\rho\:}_{0}\left({H}_{n}+1.7{R}_{n}\right)/{S}_{n}$ and $\:{K}_{1}={\rho\:}_{0}{c}_{0}^{2}/{V}_{c}$ are, respectively, the equivalent mass and stiffness of the HR, and *U*_*1*_ is the volume velocity in the neck^46^. *R*_*1*_ is the equivalent damping coefficient, but since the bulk viscosity of air is between 18.1 µPa s and 18.8 µPa s at room temperature^55,56^, *R*_*1*_ can be neglected. Membrane-HR systems described in the literature consider models where sound impinges only from one end of the structure (either the membrane or the neck) and design the experiments accordingly^46,52,53^. Nevertheless, two-port systems subject to plane wave propagation in open air will be subject to two incoming pressures – *p*_*1*_ and *p*_*2*_. By taking the centre of the membrane-coupled HR system as the geometrical reference and assuming that the wave arrives from a direction *θ* (as displayed in Fig. 5A), it can be found that *p*_*1*_ and *p*_*2*_ are given by Eq. (5) and Eq. (6):

5 $$\:{p}_{1}={p}_{0}\text{exp}\left[i\omega\:\left(t+\frac{{H}_{c}+{L^{\prime}}}{{2c}_{0}}\:\right)\text{cos}\theta\:\right]$$

6 $$\:{p}_{2}={p}_{0}\text{exp}\left[i\omega\:\left(t-\frac{{H}_{c}+{L^{\prime}}}{{2c}_{0}}\:\right)\text{cos}\theta\:\right]$$

Hence, the governing equations for the acousto-mechanical model constituted by the membrane-coupled HR system subject to plane wave excitation in open air conditions are given by:

7 $$\:\left\{{M}_{1}{\dot{U}}_{1}+{K}_{1}\int\:\left({U}_{1}-{U}_{2}\right)dt={p}_{1}\right.$$

8 $$\:\left\{{M}_{2}{\dot{U}}_{2}+{D}_{2}{U}_{2}+{K}_{2}\int\:{U}_{2}dt+{K}_{1}\int\:\left({U}_{2}-{U}_{1}\right)dt=-{p}_{2}\right.$$

where $\:{M}_{2}={M}_{eq,m}^{\left(p\right)}/{S}_{m}^{2}$, $\:{D}_{2}={D}_{eq,m}^{\left(p\right)}/{S}_{m}^{2}$, and $\:{K}_{2}={K}_{eq,m}^{\left(p\right)}/{S}_{m}^{2}$ (where *S*_*m*_ is the area of the membrane), and *U*_*2*_ is the volume velocity of the air aroused by the membrane’s first mode of vibration. The same system can be solved for a centre-mass membrane model simply by replacing $\:{M}_{eq,m}^{\left(p\right)}$, $\:{D}_{eq,m}^{\left(p\right)}$, and $\:{K}_{eq,m}^{\left(p\right)}$ for $\:{M}_{eq,m}^{\left(c\right)}$, $\:{D}_{eq,m}^{\left(c\right)}$, and $\:{K}_{eq,m}^{\left(c\right)}$.

### Finite element analysis

To verify the mathematical model proposed above, the commercial software COMSOL Multiphysics was employed to develop the corresponding model. COMSOL was utilised to perform: (i) membrane eigenfrequency studies, (ii) HR frequency domain studies, and (iii) membrane-coupled HR frequency domain studies via 2D axisymmetric geometries.

### Membrane eigenfrequency studies

The resonance frequencies of the membrane were determined by using an *Eigenfrequency* study. The outer edge of the membrane was set to 0 prescribed displacement in the *z* direction, and the tension was implemented by applying an *Initial Stress and Strain* material model, according to COMSOL’s guidelines^57^. The *Eigenfrequency* study included geometric nonlinearities in order to avoid stress artifacts, as well as a parametric sweep over tension values between 2.22 N/m and 19.98 N/m in steps of 2.22 N/m. Six eigenfrequency values were searched around 1 Hz.

### Helmholtz resonator frequency domain studies

The resonance of the HR was determined by using a *Frequency Domain* study and sweeping from 50 Hz to 15 kHz in steps of 100 Hz. The *Solid Mechanics*, *Pressure Acoustics*, and *Thermoviscous Acoustics* modules were employed with their corresponding coupled physics. The *Thermoviscous Acoustics* module was employed for the air domain inside the HR to account for the thermal and viscous losses near the walls – an effect that is more pronounced at resonance. The overall pressure inside the HR was determined by defining an *Integration Nonlocal Coupling* in the HR air domain (to define its total volume), and integrating the thermal pressure over the HR’s volume. Finally, the SPL was calculated with respect to the incoming pressure and plotted against frequency.

### Membrane-coupled Helmholtz resonators frequency domain studies

The resonance of the membrane-HR AMMs was determined analogously to the process described in the *Helmholtz resonator frequency domain studies* section, with the addition of the *Membrane* module. The bottom rigid wall of the HR was substituted by a membrane with the same boundary conditions described in *Membrane eigenfrequency studies*. Moreover, *Face Load* boundary conditions were added on the membrane’s surface to ensure correct loading of the incoming pressure wave. In this case, two additional Multiphysics modes were required to ensure *Acoustic-Membrane* and *Thermoviscous-Membrane* interactions. The mesh was consequently adjusted to account for 20 boundary layers at a size determined by the resonance frequency of the HR (which is the highest and will, therefore, provide a smaller boundary layer size)^58^. The frequency range was adjusted to solve with higher resolution (from 50 Hz to 15 kHz in steps of 25 Hz).

### Fabrication of acoustic metamaterials

The HR AMM samples (Fig. 5B) were fabricated using a PRUSA SL1S 3D printer (Prague, Czech Republic) equipped with a 405 nm LED light source. The samples were designed via computer aided design (CAD) in Autodesk Inventor and exported as STL files, which were posteriorly imported into PRUSASlicer. The STL files were then sliced at 100 μm layer thickness without any supports nor pads using the settings for the commercially available PRUSA Deep Blue Transparent Tough (PRUSA) resin, with predefined exposure time per layer of 10 s, and initial exposure time of 25 s, leading to fabrication times of 30 min. After 3D-printing, the samples were thoroughly washed in isopropyl alcohol under sonication until all excess resin was removed. Removal of resin trapped within the HRs was aided by using a compressed air gun. Finally, the samples were UV post-processed for 10 min in the PRUSA Curing and Washing Machine to ensure crosslinking of any residual uncured functional groups. For the membrane-coupled HR AMMs, base-free HRs were fabricated following the same process described above. After 3D-printing and post-processing, 60 μm nitrile membranes were pre-stretched and attached to the based of the 3D-printed HRs, and covered with 3D-printed rings to ensure tension on the membrane (Fig. 5C). Four sets of tensions (labelled from *T1* to *T4*) were prepared following this approach, leading to a total of 35 samples.

### Determination of membrane tension

The tension on the membranes was determined by using a 3D laser Doppler vibrometer (LDV) system with an MSA-100-3D scanning head (Polytec, Waldbronn, Germany). The internal function generator of the LDV was employed to drive a piezoelectric stack using a periodic chirp from 50 Hz to 10 kHz at 1 V peak-to-peak amplitude, and with an offset of 0.5 V. A circular region covering the full area of the membrane was defined with a total of 257 scanned points, and the measurements were externally TTL triggered (internally synchronised with the electric signal) with a positive slope and without pre-triggering. Each point of the scan consisted of 800 FFT lines, leading to a sample time of 80 ms, and a resolution of 12.5 Hz. This set up enabled determination of the characteristic frequencies of the membranes, and the fundamental frequency was used to determine *T*_*s*_ via Eq. (9)^59^.

9 $$\:\omega\:=\frac{2.4048}{{R}_{m}}\sqrt{\frac{{T}_{s}}{{h}_{m}{\rho\:}_{m}}}$$

where *h*_*m*_ and *ρ*_*m*_ are, respectively, the thickness and density of the membrane, and 2.4048 is the first root of order 0 of the Bessel function^60^.

### Acoustic testing

**Fig. 6:**
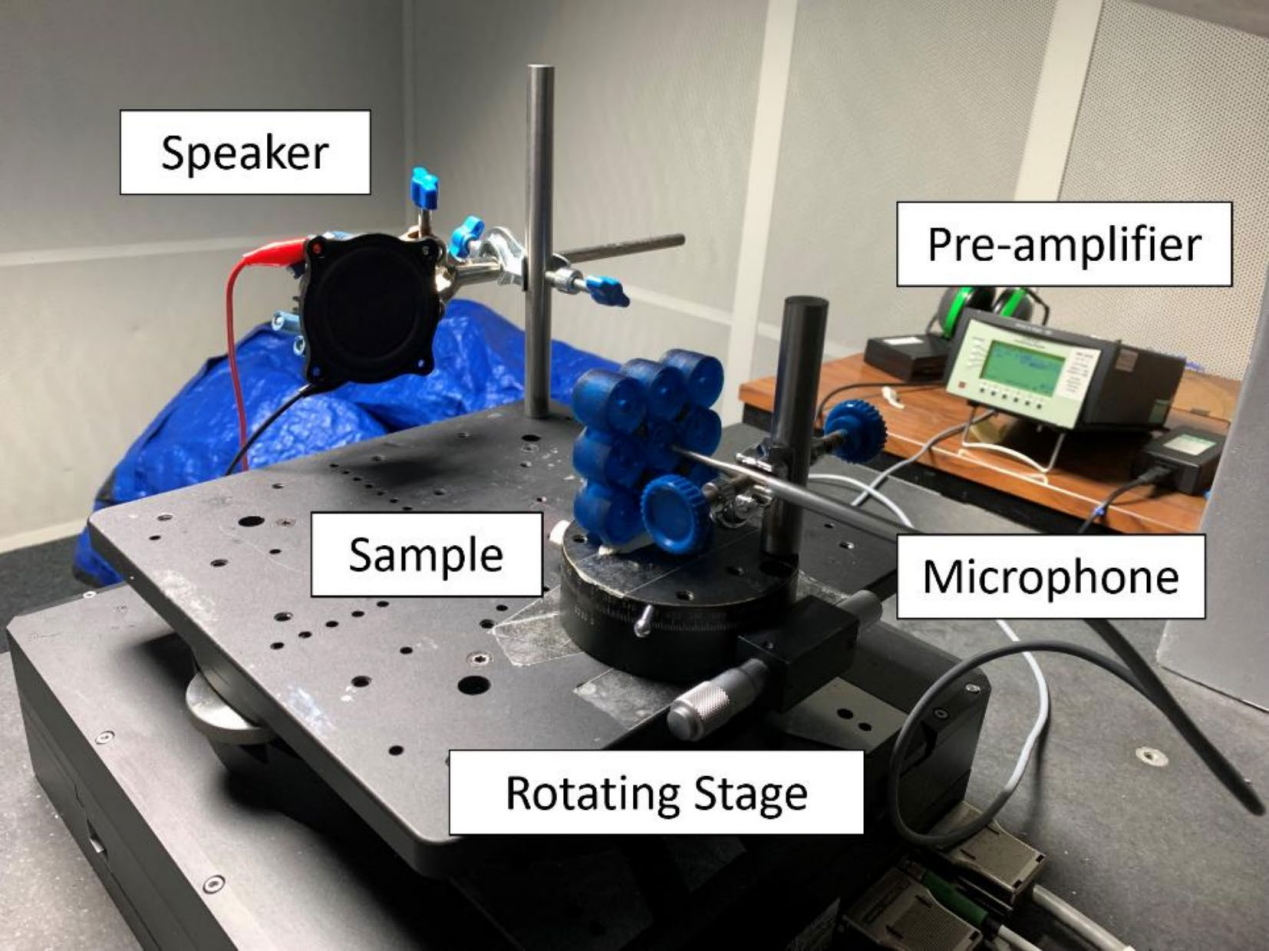
Experimental set up. The sample and the microphone are mounted on a rotating stage such that they both move relative to the speaker. The speaker and the sample-microphone system sit on an anti-vibration table inside an acoustic booth to minimise noise – particularly relevant at low frequencies. The speaker is connected to the laser Doppler vibrometer data acquisition system signal generator, and the microphone is connected to the pre-amplifier.

The LDV data acquisition system was employed to record the acoustic response of the AMMs. The structures were mounted on a rotating stage and placed in front of a loudspeaker (Visaton WS 17 E), with the bottom part of the HR initially facing the speaker (Fig. 6) – this position will be referred to as 0° position. Analogously to the set up described in the previous section, the internal signal generator of the LDV was used to drive the acoustic signal (periodic chirp from 50 Hz to 15 kHz) through an acoustic amplifier (Onkyo A-9010) connected to the loudspeaker. The acoustic response was acquired via a Bruel & Kjaer (B&K) 1/8-inch pressure field microphone (4138-A-015, Naerum, Denmark) connected to a pre-amplifier (WH-3219) and positioned directly next to the neck of the HR. The microphone-pre-amplifier system was externally TTL triggered (internally synchronised with the acoustic signal) with a positive slope and without pre-triggering. Prior to any measurements, the acoustic field was flattened via a correction file to ensure that the measured acoustic response corresponds to the AMMs rather than to the inherent characteristics of the microphone. Each measurement consisted of 16 complex averages and 6400 FFT lines, leading to sample times of 512 ms ms and resolutions of 1.95 Hz. Acoustic measurements were taken from 0° to 360° in steps of 5°.

## Electronic supplementary material

Below is the link to the electronic supplementary material.

Supplementary Material 1

## Data Availability

The datasets generated and/or analysed during the current study are not publicly available due to the data being part of ongoing research but are available from the corresponding author on reasonable request.
