# Supplementary material for: On the directionality of membrane coupled Helmholtz resonators under open air conditions
Source: Sci Rep. 2024 Nov 13;14:27771. doi: 10.1038/s41598-024-79568-9 (PMC11561294; doi:10.1038/s41598-024-79568-9)
Supplement: Supplementary file 1 — Supplementary Material 1 [file 41598_2024_79568_MOESM1_ESM.docx]

**On the directionality of membrane-coupled Helmholtz resonators under open-air conditions**

R. Domingo-Roca^a^*, A. Feeney^b^, J. F. C. Windmill^a^, J. C. Jackson-Camargo

^a^ Centre for Ultrasonic Engineering, Electronic & Electrical Engineering, University of Strathclyde.

^b^ Centre for Medical Ultrasound, James Watt School of Engineering, University of Glasgow.

* corresponding author: [roger.domingo-roca@strath.ac.uk](mailto:roger.domingo-roca@strath.ac.uk)

**1. X-ray micro-computed tomography**

The morphology of the 3D-printed acoustic metamaterials (AMMs) was investigated using micro-computed tomography (μCT, Bruker Skyscan, 1172, Belgium) at 6 μm isotropic voxel size. The scan was performed using 50 kVp tube voltage, 100 μA tube current, 829 ms exposure time, 0.2^o^ rotation step (for a total of 360^o^), with frame averaging of 3, and with an Al-Cu filter. The acquired data was reconstructed using Skyscan NRecon software (Bruker, Version 1.6.9.18). Prior to three-dimensional representation, a task list was created in CTAn software (Bruker, version 1.20.8) to automatically generate a volume of interest to serve as the total volume to berepresented, including thresholding between 3 and 255, and two steps of despeckling; one to remove black speckles (below 100 μm in 2D space), and another one to remove white speckles (below 50 μm), defined to take place on each Image.

**2. Laser Doppler vibrometry: membrane surface representation**

| 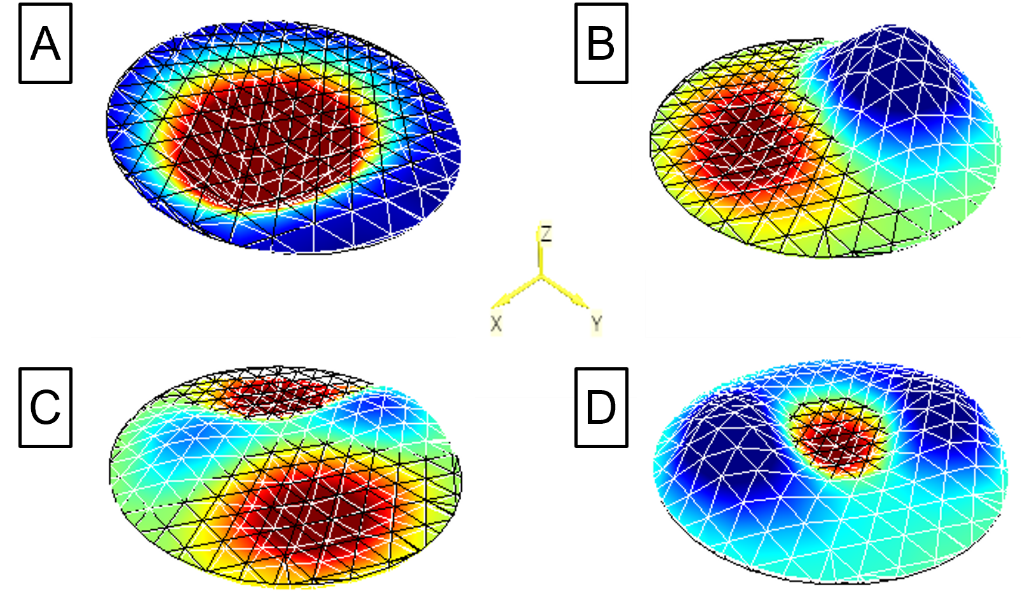  **Fig. S1:** Three-dimensional representation of the surface motion of one of the membranes scanned via 3D laser Doppler vibrometry (LDV) under excitation via a piezoelectric stack. (A) (0,1) mode, 700 Hz, (B) (1,1) mode, 2163 Hz, (C) (2,1) mode, 2650 Hz, and (D) (0,2) mode, 3238 Hz. |
| --- |

A representative 3D laser Doppler vibrometry (LDV) surface scan of one of the pre-stretched membranes is shown in Supplementary Figure S1, including the first four eigenmodes – (0,1), (1,1), (2,1), and (0,2) – and their corresponding eigenfrequencies. The membranes of all the membrane-coupled HR AMMs were scanned prior to experimentation and their corresponding tension values were determined from Eq. (8) using the frequency of the fundamental mode.

**3. Determination of membrane tension**

**Supplementary Table I:** Frequencies (in Hz) of the fundamental mode for each tested membrane and their corresponding tension value (in N/m).

| ***T1* subset** | | | | ***T2* subset** | | |
| --- | --- | --- | --- | --- | --- | --- |
| **Position** | **Frequency (Hz)** | **Tension (N/m)** | | **Position** | **Frequency (Hz)** | **Tension (N/m)** |
| 1 | 900.0 | 11.96 | 1 | | 588.8 | 4.61 |
| 2 | 1100.0 | 17.86 | 2 | | 700.0 | 7.23 |
| 3 | 752.5 | 8.36 | 3 | | 663.8 | 6.50 |
| 4 | 700.0 | 7.23 | 4 | | 900.0 | 11.96 |
| 5 | 688.0 | 6.99 | 5 | | 853.8 | 10.76 |
| 6 | 938.0 | 12.99 | 6 | | 875.0 | 11.30 |
| 7 | N/A | N/A | 7 | | 538.8 | 4.29 |
| 8 | 623.8 | 5.74 | 8 | | 477.5 | 3.37 |
| 9 | 800.0 | 9.45 | 9 | | 722.5 | 7.71 |
| ***T3* subset** | | | ***T4* subset** | | | |
| **Position** | **Frequency (Hz)** | **Tension (N/m)** | **Position** | | **Frequency (Hz)** | **Tension (N/m)** |
| 1 | 900.0 | 13.95 | 1 | | 765.0 | 10.08 |
| 2 | 992.5 | 16.97 | 2 | | 1043.8 | 18.77 |
| 3 | 960.0 | 15.88 | 3 | | 1000.0 | 17.23 |
| 4 | 1147.5 | 22.68 | 4 | | 1018.8 | 17.88 |
| 5 | 1018.8 | 17.88 | 5 | | 1000.0 | 17.23 |
| 6 | 800.0 | 11.02 | 6 | | 1040 | 18.63 |
| 7 | 803.8 | 11.13 | 7 | | 1000.0 | 17.23 |
| 8 | 787.5 | 10.68 | 8 | | 972.5 | 16.29 |
| 9 | 941.3 | 15.26 | 9 | | 1084.4 | 20.26 |

**4. Values of the bandgap frequency and acoustic attenuation of the membrane-HR AMMs**

**Supplementary Table II:** Experimental and theoretical bandgap frequency (BGF) values (in Hz) obtained as a function of applied membrane tension.

| **T1 subset** | | | **T2 subset** | | |
| --- | --- | --- | --- | --- | --- |
| **Position** | **Tension (N/m)** | **Experimental BGF (Hz)** | **Position** | **Tension (N/m)** | **Experimental BGF (Hz)** |
| 1 | 11.96 | 814.32 | 1 | 4.61 | 587.50 |
| 2 | 17.86 | 1262.50 | 2 | 7.23 | 729.95 |
| 3 | 8.36 | 861.46 | 3 | 6.50 | 702.86 |
| 4 | 7.23 | 753.73 | 4 | 11.96 | 889.84 |
| 5 | 6.99 | 702.60 | 5 | 10.76 | 956.25 |
| 6 | 12.99 | 952.60 | 6 | 11.30 | 959.38 |
| 7 | N/A | N/A | 7 | 4.29 | 699.69 |
| 8 | 5.74 | 756.25 | 8 | 3.37 | 625.26 |
| 9 | 9.45 | 809.64 | 9 | 7.71 | 883.33 |
| ***T3* subset** | | | ***T4* subset** | | |
| **Position** | **Tension (N/m)** | **Experimental BGF (Hz)** | **Position** | **Tension (N/m)** | **Experimental BGF (Hz)** |
| 1 | 13.95 | 1250.78 | 1 | 10.08 | 901.56 |
| 2 | 16.97 | 1228.13 | 2 | 18.77 | 1167.19 |
| 3 | 15.88 | 1179.43 | 3 | 17.23 | 1143.23 |
| 4 | 22.68 | 1212.24 | 4 | 17.88 | 1381.77 |
| 5 | 17.88 | 1130.47 | 5 | 17.23 | 1212.50 |
| 6 | 11.02 | 938.28 | 6 | 18.63 | 1136.72 |
| 7 | 11.13 | 1062.50 | 7 | 17.23 | 1192.45 |
| 8 | 10.68 | 955.73 | 8 | 16.29 | 989.06 |
| 9 | 15.26 | 1091.67 | 9 | 20.26 | 1315.63 |
| **Theoretical results** | | | | | |
| **Tension (N/m)** | **FEA** | **Piston model** | | **Centre-mass model** | |
| 2.22 | 450 | 326.34 | | 436.35 | |
| 4.44 | 580 | 458.84 | | 615.87 | |
| 6.66 | 710 | 558.66 | | 752.67 | |
| 8.88 | 820 | 641.26 | | 867.11 | |
| 11.10 | 910 | 712.65 | | 967.02 | |
| 13.32 | 1000 | 775.95 | | 1056.44 | |
| 15.54 | 1080 | 832.99 | | 1137.71 | |
| 17.76 | 1160 | 885.03 | | 1212.34 | |
| 19.98 | 1230 | 932.90 | | 1281.36 | |
| 30 | 1510 | 1110.98 | | 1538.42 | |
| 40 | 1740 | 1246.24 | | 1724.23 | |
| 50 | 1840 | 1353.39 | | 1851.46 | |

**Supplementary Table III:** Attenuation (in dB) of each of the investigated membrane-HR AMMs as a function of applied membrane tension.

| ***T1* subset** | | | ***T2* subset** | | | | |  |
| --- | --- | --- | --- | --- | --- | --- | --- | --- |
| **Position** | **Tension (N/m)** | **Attenuation (dB)** | **Position** | **Tension (N/m)** | | **Attenuation (dB)** | |  |
| 1 | 11.96 | 3.30 | 1 | | 4.61 | | 8.13 | |
| 2 | 17.86 | 37.07 | 2 | | 7.23 | | 12.86 | |
| 3 | 8.36 | 15.75 | 3 | | 6.50 | | 7.87 | |
| 4 | 7.23 | 8.80 | 4 | | 11.96 | | 10.49 | |
| 5 | 6.99 | 10.96 | 5 | | 10.76 | | 14.94 | |
| 6 | 12.99 | 10.07 | 6 | | 11.30 | | 7.38 | |
| 7 | N/A | N/A | 7 | | 4.29 | | 9.10 | |
| 8 | 5.74 | 7.44 | 8 | | 3.37 | | 18.04 | |
| 9 | 9.45 | 6.08 | 9 | | 7.71 | | 15.52 | |
| ***T3* subset** | | | ***T4* subset** | | | | | |
| **Position** | **Tension (N/m)** | **Attenuation (dB)** | **Position** | | **Tension (N/m)** | | **Attenuation (dB)** | |
| 1 | 13.95 | 10.11 | 1 | | 10.08 | | 1.80 | |
| 2 | 16.97 | 6.29 | 2 | | 18.77 | | 3.58 | |
| 3 | 15.88 | 6.93 | 3 | | 17.23 | | 10.54 | |
| 4 | 22.68 | 13.20 | 4 | | 17.88 | | 5.10 | |
| 5 | 17.88 | 28.83 | 5 | | 17.23 | | 7.83 | |
| 6 | 11.02 | 7.18 | 6 | | 18.63 | | 8.89 | |
| 7 | 11.13 | 3.89 | 7 | | 17.23 | | 3.62 | |
| 8 | 10.68 | 7.57 | 8 | | 16.29 | | 3.18 | |
| 9 | 15.26 | 8.38 | 9 | | 20.26 | | 5.43 | |

Cells labelled as ‘N/A’ indicate breakage of the membrane.

**5. Directional response**

**5.1. Directional response of the HR AMM (one-port acoustic system)**

| 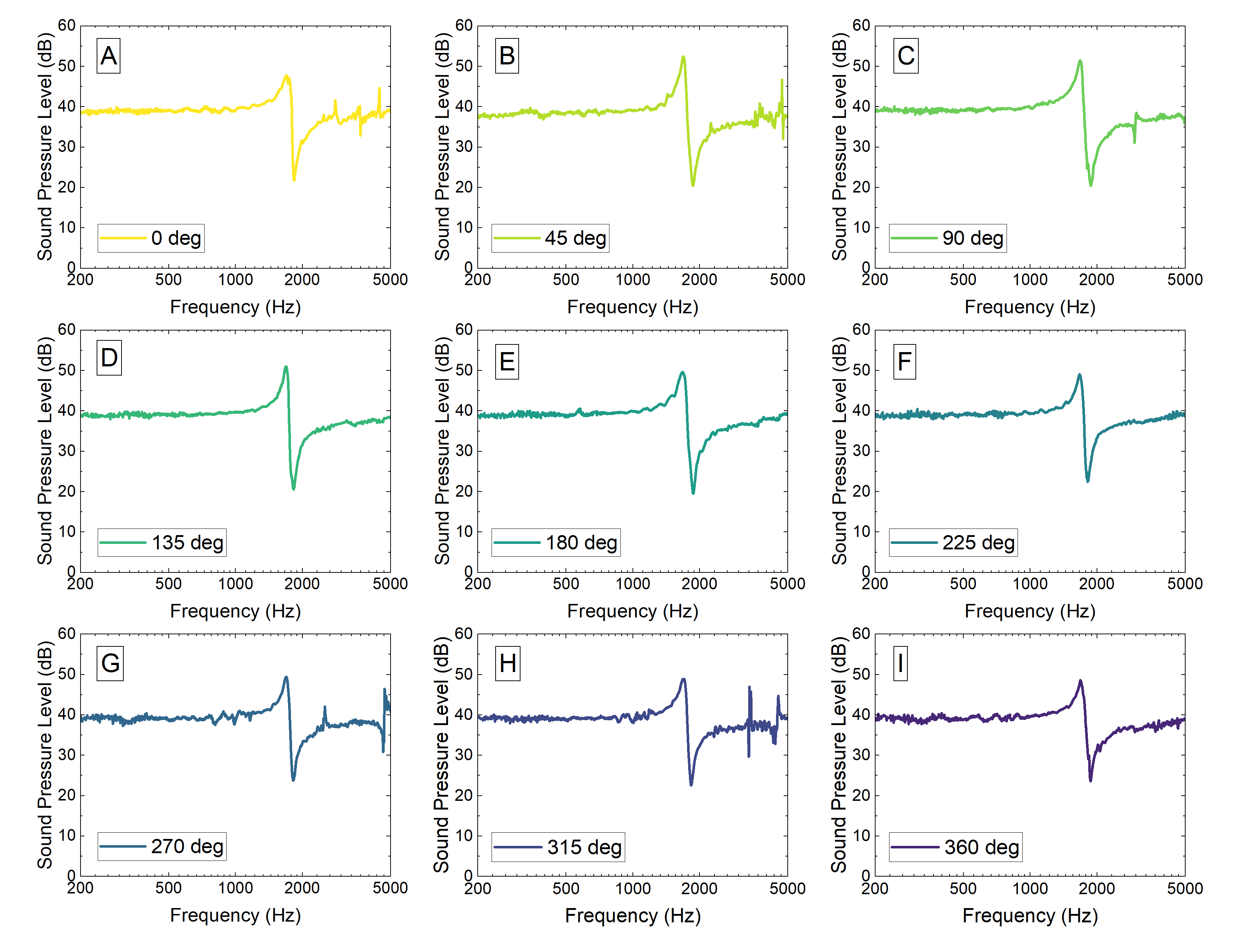  **Fig. S2:** Sound pressure level (SPL) recorded at the neck of the HR AMM from 200 Hz to 5,000 Hz at (A) 0^o^, (B) 45^o^, (C) 90^o^, (D) 135^o^, (E) 180^o^, (F) 225^o^, (G) 270^o^, (H) 315^o^, and (I) 360^o^. |
| --- |

| 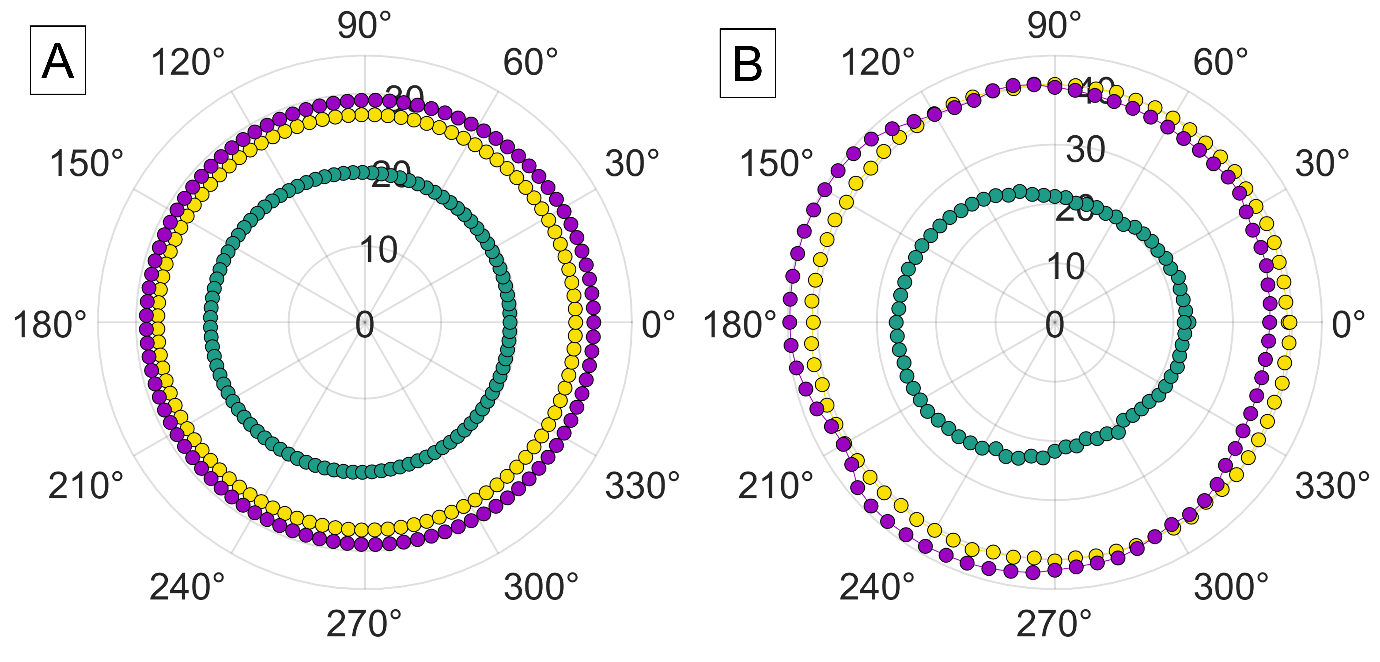  **Fig. S3:** (A) and (B) show, respectively, the theoretical and experimental directional response of the Helmholtz resonator (HR) acoustic metamaterial (AMM) at 500 Hz (yellow), 1850 Hz (green), and 3,000 Hz (purple). |
| --- |

**5.2. Directional response of membrane-coupled HR AMMs: COMSOL**

| 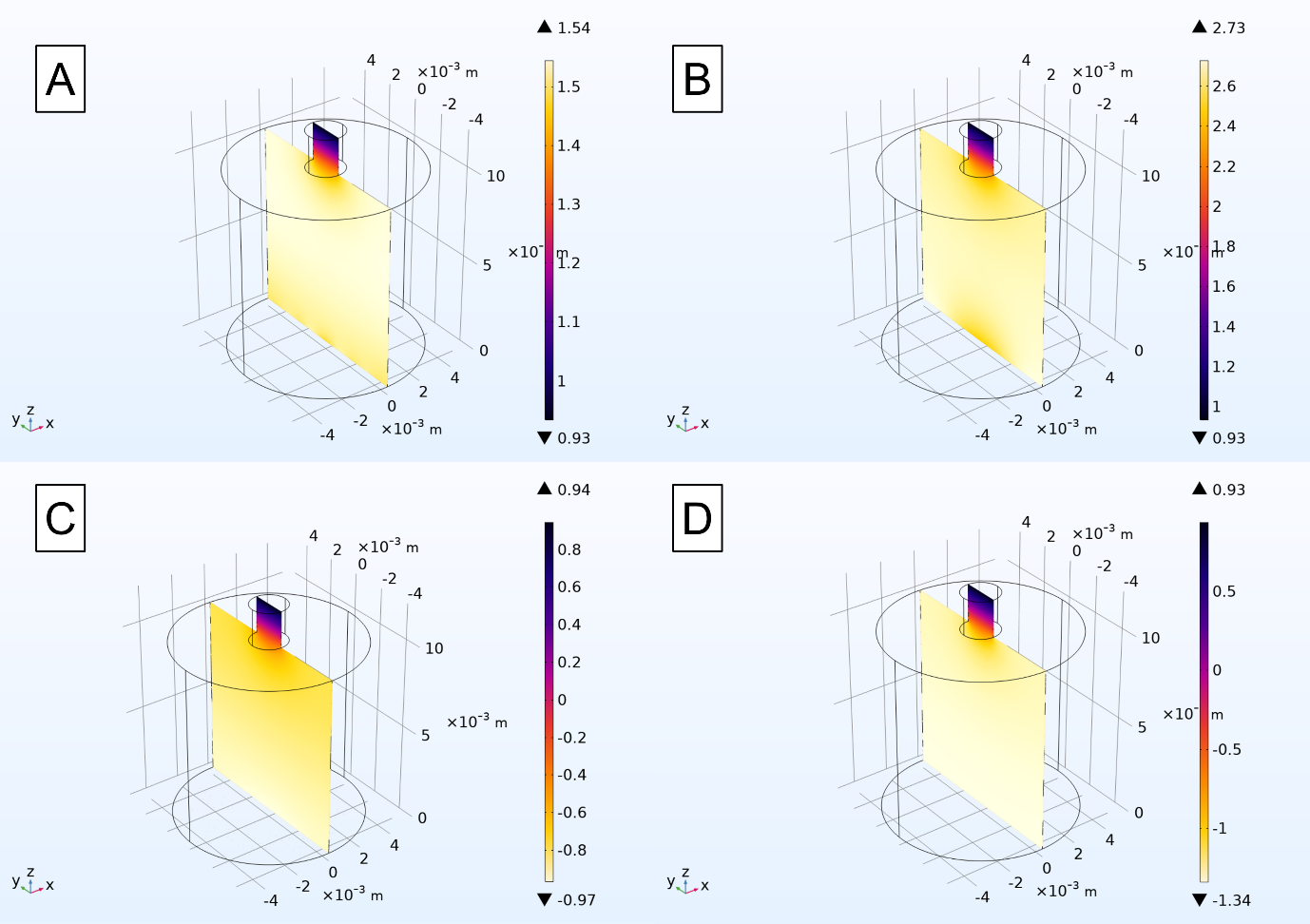  **Fig. S4:** Finite element analysis (FEA) of the total pressure (in Pa) (A)-(c) inside of the membrane-HR AMM with a membrane under tension (T) values of 1 N/m, 30 N/m, and 100 N/m, respectively, and (D) inside the Helmholtz resonator (HR) acoustic metamaterial (AMM) |
| --- |

**5.3. Directional response of membrane-coupled HR AMMs: Experimental influence of resonator size**

| 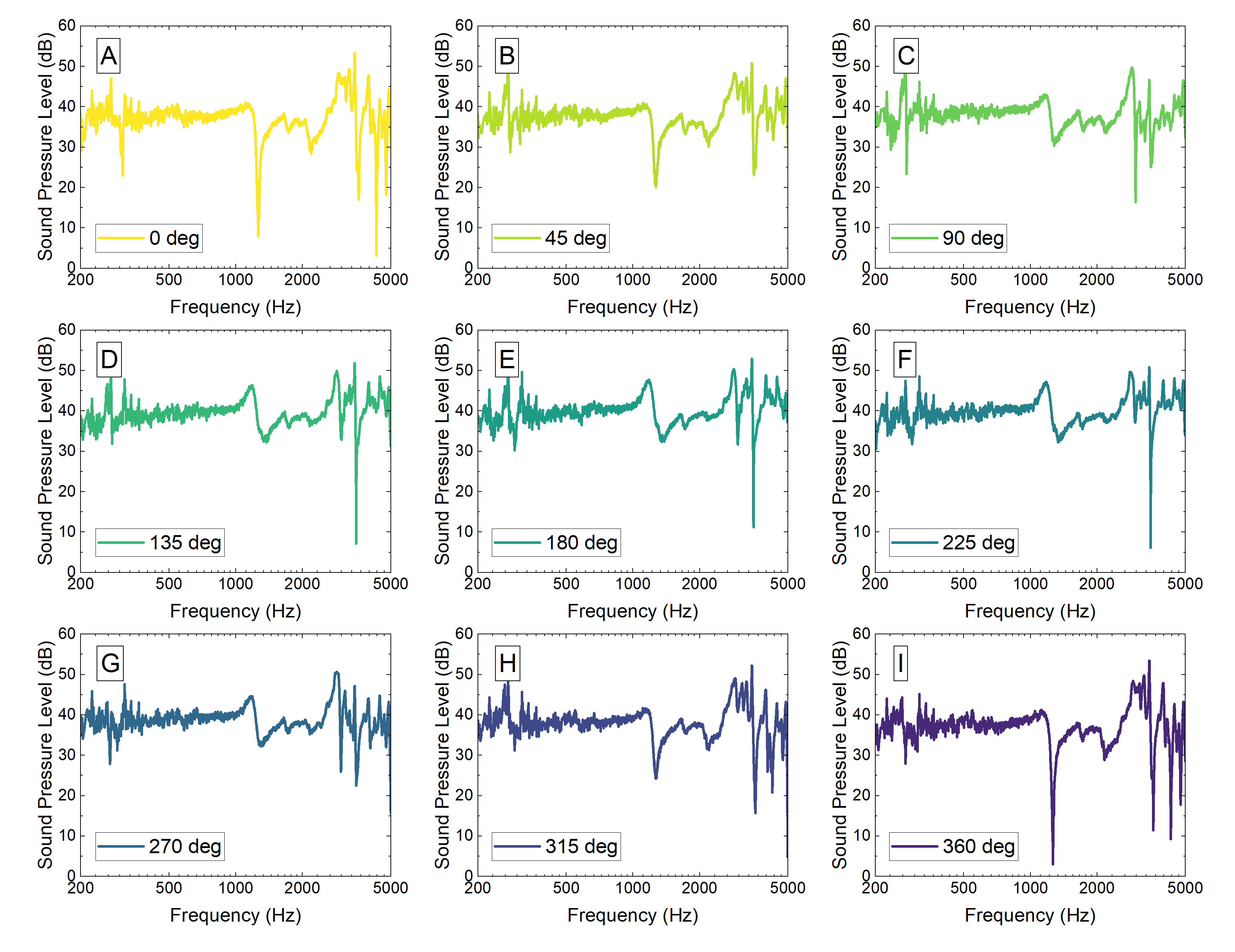  **Fig. S5:** Sound pressure level (SPL) recorded at the neck of the membrane-HR AMM from 200 Hz to 5,000 Hz at (A) 0^o^, (B) 45^o^, (C) 90^o^, (D) 135^o^, (E) 180^o^, (F) 225^o^, (G) 270^o^, (H) 315^o^, and (I) 360^o^. |
| --- |

|  |
| --- |

| 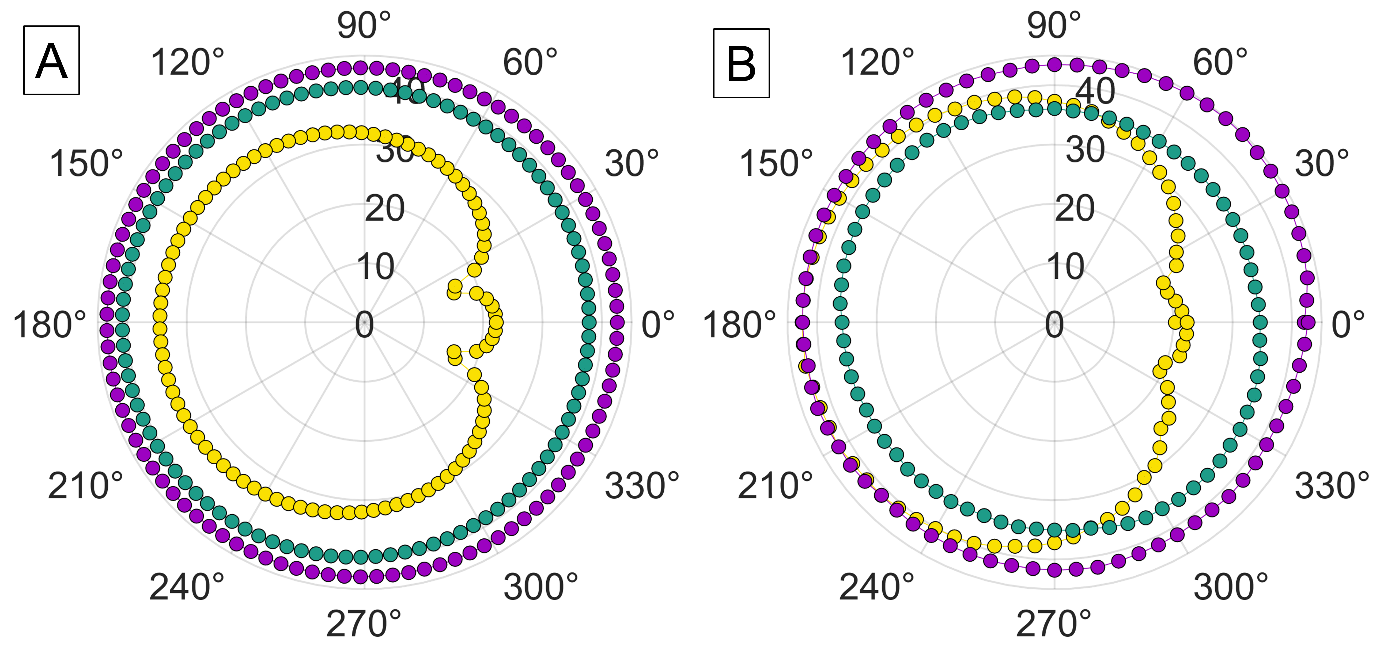  **Fig. S6:** (A) and (B) show, respectively, the directional response of a membrane-Helmholtz resonator system at the bandgap frequency (702 Hz, in yellow), and its first two harmonics (1404 Hz and 2106 Hz in green and purple, respectively). |
| --- |
| 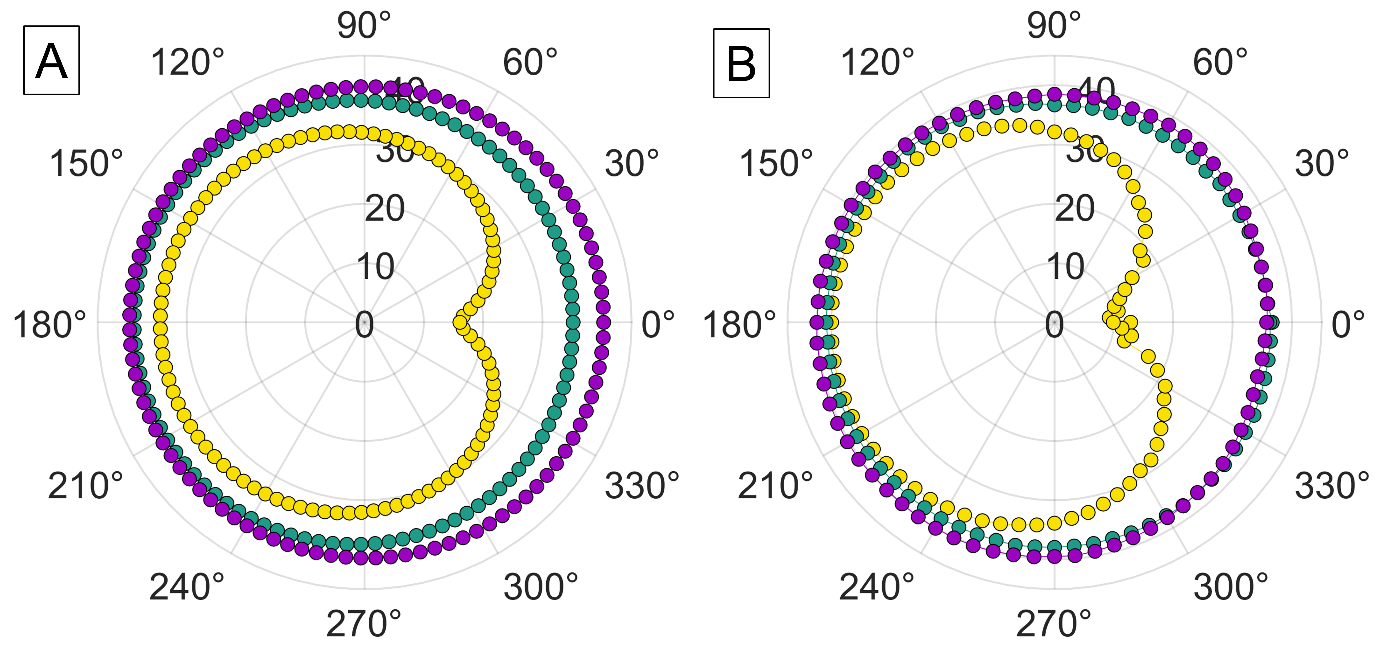  **Fig. S7:** (A) and (B) show, respectively, the theoretical and experimental acoustic response (in dB) of a membrane-Helmholtz resonator acoustic metamaterial (AMM) with H_c_ = 5 mm, H_n_ = 1 mm, R_c_, = 5 mm, R_n_ = 1 mm, and R_m_ = 5 mm under membrane tension of 20 N/m |

**5.4. Directional response of the membrane**

| 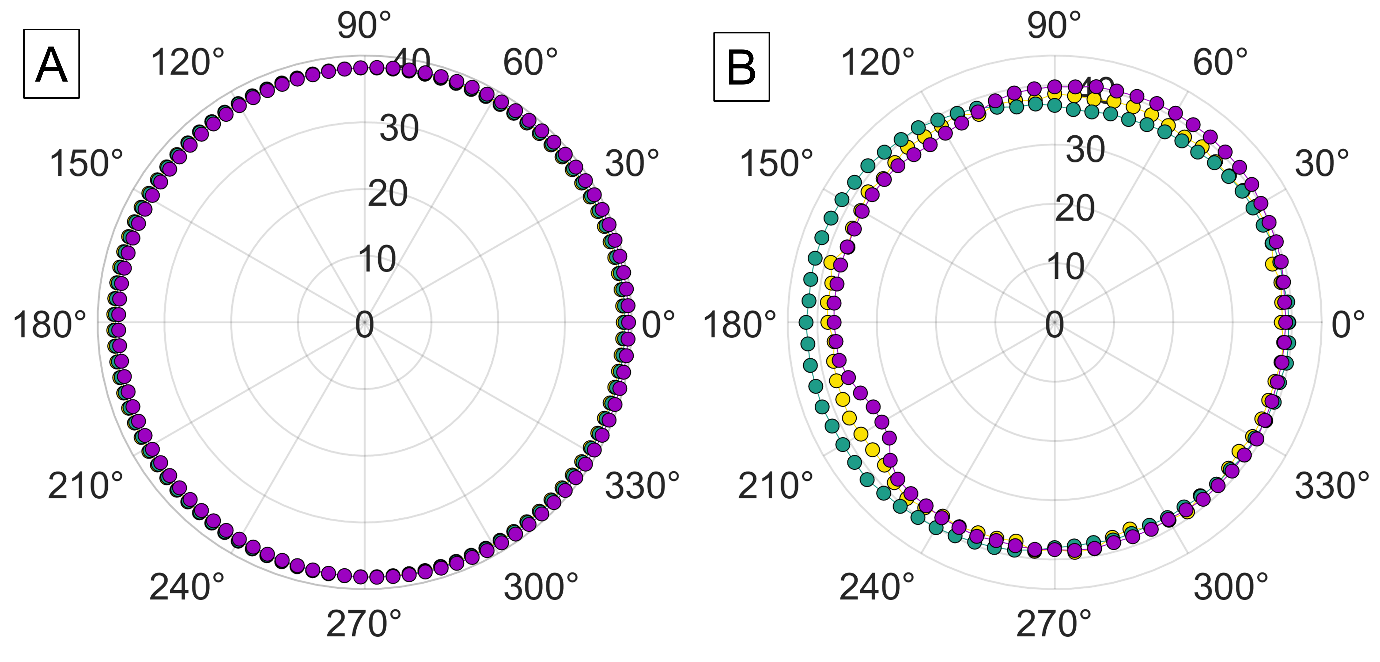  **Fig. S8:** (A) and (B) show, respectively, the directional response of a membrane at its fundamental eigenfrequency (727 Hz, yellow), 1850 Hz (green), and 3000 Hz (purple). |
| --- |

**6. Piston vs centre-mass model**

| **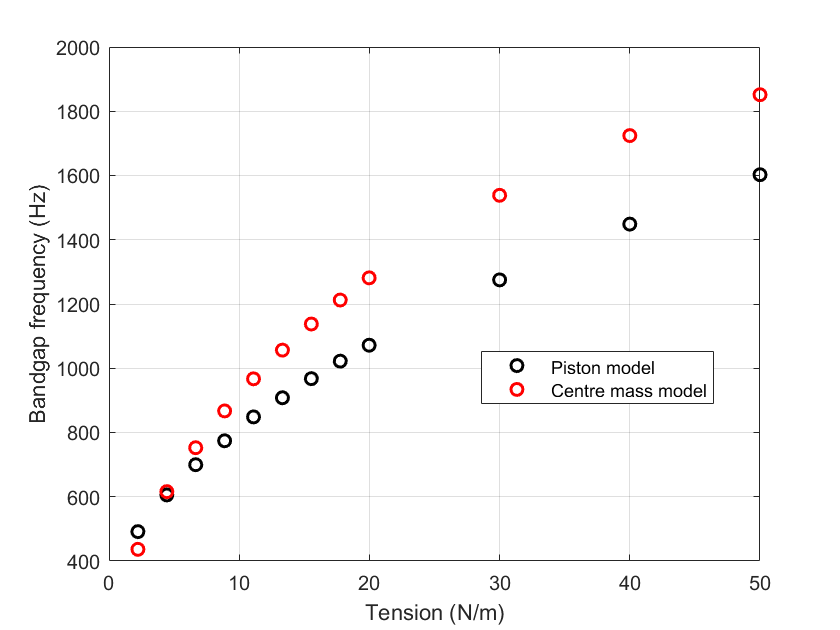**  **Fig. S9:** Bandgap frequency of the membrane-coupled Helmholtz resonator (HR) acoustic metamaterial (AMM) with increasing membrane tension under the piston (black) and centre-mass (red) assumptions. |
| --- |
